# Notes and News

**Published:** 1899-08-12

**Authors:** 


### NOTES AND NEWS.

(Collected and Communicated.)

The Chelsea Hospital for Women has received £100 from the executors of the late Mr. John Bradley.

The opening meeting of the Otological Congress took place on Tuesday at the Examination Hall, Victoria Embankment. Professor Urban Pritchard, the president-elect, was in the chair, and there were present about 300 aural surgeons from various parts of the world.

In answer to questions in the House of Commons, Mr. T. W. Russell stated that the demand for lymph had very largely increased, until in one week it had reached 2,287 tubes a day. During the last two months it had averaged 1,790 tubes a day. He could not at present make any promise as to the supply of the lymph for the use of private practitioners.

Mr. Broadhurst, in calling attention to the Leicester vaccination question in the House of Commons, remarked that, if the Local Government Board persisted, 45 ladies and gentlemen would go to prison on the present occasion, to be joined by their successors, until the whole population would ultimately have to be accommodated. Mr. Chaplin pointed out that the guardians by their action were depriving the people of facilities. As a matter of fact the number requiring vaccination was larger now than before the compulsory clause was removed from the Act. In the first six months of 1898 734 cases were vaccinated; in the first six months of 1899 1,977 individuals were vaccinated.

According to the most recently issued report of the Army Medical Department (which is for 1897 !) typhoid fever in India caused 2,050 admissions, 556 deaths, and a constant sickness of 296·85 during the year, being in the ratio of 31·8, 8·62, and 4·60 per 1,000 respectively; thus nearly half of all the deaths which occurred among British troops in India were caused by typhoid fever. In some of the stations the disease has evidently become endemic, and it seems as if it were generally caught in the native drink shops and other objectionable institutions rather than in the barracks. The youth new to the country is far more liable than his more experienced fellow to catch the disease, by far the greater number both of admissions and deaths occurring among men who have been less than one year in the country. More than half a full battalion carried off each year by one "preventable" disease!

Dr. Starling, F.R.S., has been appointed Professor of Physiology at University College in place of Professor Schafer. Dr. Starling holds the post of Lecturer in Physiology at Guy's Hospital.

The example of England in relaxing the vaccination laws has not made a favourable impression abroad. In Japan the government is about to enforce, not only vaccination but revaccination also. Children are to be vaccinated at ten months, at six, and at twelve years of age.

The overcrowding in St. Pancras was the subject of some discussion at the last meeting of the London County Council, and a proposal is before that body to report the vestry to the Local Government Board as failing in providing sufficient sanitary inspectors. Owing to the large amount of business before the Council the subject was adjourned. By way of defence the vestry announce that they have appointed more inspectors, and that the overcrowding is beyond their control.

The annual dinner of the British Medical Association at Portsmouth was a very successful function. About 300 were present, and at the same time a ladies' reception was given by the Mayoress and ladies of the local committee.

On Friday Dr. Newsholme, of Brighton, opened a discussion on the efficiency of existing measures directed against the spread of infection in elementary schools. The subject is a wide one, and it was well discussed in its many bearings, and it was suggested that a resolution should be passed in favour of giving medical officers the right to enter schools for purposes of inspection. In the end, a resolution was passed asking the Council to take action in the matter.

Temperance has been well to the fore in Portsmouth during the week, a public meeting having been held, and a temperance breakfast given by the league to members of the British Medical Association. It was too hot, however, to take things very seriously, so we will not take too seriously the statement made by one gentleman that he found it very hard indeed to be a total abstaining doctor, "in fact, he might double his practice by following the example of other doctors!" nor that made by a northern professor (who, it is said, secured attention by his humorous stories given in a broad Scotch accent) to the effect that, notwithstanding the number of his operations, "his death rate was so low that the pathologist grumbled at him."

At the concluding general meeting held on Friday, at which there was but a very small attendance, certain resolutions sent up by the sections were adopted. It was, however, only a formal affair. One of these resolutions urged that witnesses giving evidence concerning "colour signals" should themselves be tested as to their sense of colour; another thanked Mr. Chamberlain for the interest he had taken in establishing the school of tropical medicine, and the third was in favour of legislation for well-to-do inebriates. Then came the usual votes of thanks, and except the ball and the excursion, all of which passed off admirably, the meeting was at an end. The Association meets next year at Ipswich.

